# Caspase-14—From Biomolecular Basics to Clinical Approach. A Review of Available Data

**DOI:** 10.3390/ijms22115575

**Published:** 2021-05-25

**Authors:** Agnieszka Markiewicz, Dawid Sigorski, Mateusz Markiewicz, Agnieszka Owczarczyk-Saczonek, Waldemar Placek

**Affiliations:** 1 Department and Clinic of Dermatology, Sexually Transmitted Diseases and Clinical Immunology, University of Warmia and Mazury, 10-229 Olsztyn, Poland; agnieszka.owczarczyk@uwm.edu.pl (A.O.-S.); waldemar.placek@uwm.edu.pl (W.P.); 2 Department of Oncology, University of Warmia and Mazury, 10-228 Olsztyn, Poland; dawid.sigorski@uwm.edu.pl; 3 Department of Ophthalmology, University of Warmia and Mazury, 10-561 Olsztyn, Poland; mateusz.markiewicz@uwm.edu.pl

**Keywords:** caspase 14, epidermis biology, cornification, pathobiology, non-apoptotic caspase

## Abstract

Caspase-14 is a unique member of the caspase family—a family of molecules participating in apoptosis. However, it does not affect this process but regulates another form of programmed cell death—cornification, which is characteristic of the epidermis. Therefore, it plays a crucial role in the formation of the skin barrier. The cell death cycle has been a subject of interest for researchers for decades, so a lot of research has been done to expand the understanding of caspase-14, its role in cell homeostasis and processes affecting its expression and activation. Conversely, it is also an interesting target for clinical researchers searching for its role in the physiology of healthy individuals and its pathophysiology in particular diseases. A summary was done in 2008 by Denecker et al., concentrating mostly on the biotechnological aspects of the molecule and its physiological role. However, a lot of new data have been reported, and some more practical and clinical research has been conducted since then. The majority of studies tackled the issue of clinical data presenting the role of caspase in the etiopathology of many diseases such as retinal dysfunctions, multiple malignancies, and skin conditions. This review summarizes the available knowledge on the molecular and, more interestingly, the clinical aspects of caspase-14. It also presents how theoretical science may pave the way for medical research. Methods: The authors analyzed publications available on PubMed until 21 March 2021, using the search term “caspase 14”.

## 1. Introduction

The subtle balance between cell death and growth in tissues plays a crucial role in the well-being of organisms. Such processes are regulated by certain proteins, many of which have been the subject of the interest of numerous scientific projects up to date. A lot of research has been done, especially on the possible ways of cell death, including necroptosis [1], pyroptosis [2], autophagy, mitotic catastrophe, and the best elucidated—apoptosis [3]. The clinical consequences of the dysfunction of particular proteins in the processes are of great concern to human health. Caspases constitute one of the groups of proteins that participate in homeostasis [4].

Caspases are proteins that play an important role in the complex cascade of reactions leading to cell apoptosis, the initiation of inflammation, or terminal cell differentiation [5,6,7]. The most important element of controlling the activity of those processes is their production in the form of inactive molecules—zymogens. They may by activated in the process of dimerization only if adequately triggered or via the production of multimolecular complexes [8,9]. The membership in the caspase protein family is defined by sequence homology with Ced-3, an important effector protein in programmed cell death in *Caenorhabditis elegans* [10].

The whole family of caspases may be divided into the following groups: those which participate in apoptosis in mammals (caspase-3, -6, -7, -8, and -9) and those which control inflammation (caspase-1, -4, -5, and -12). Caspases -2, -12, and -14 have a more complex function, so they cannot be included in any of those groups [11].

Apoptosis is a form of cell death. It involves the destruction of the intracellular structures without inflammation and damage to other cells in the vicinity. Some caspases may initiate the process (caspase-8 and -9), and some are effector enzymes (caspase-3, -6, and -7). The presence of long or short N-terminal pro-domains is an important feature of caspases. Depending on their reaction in the apoptosome, long prodomain caspases initiate caspase activation by cleaving short prodomain caspases, which are considered executioner caspases. Caspases -1, -2, -4, -5, -8, -9, -10, -11, -12, and -13 contain long pro-domains, whereas caspases -3, -6, -7, and -14 are characterized by short pro-domains [11].

Some caspases play an important role in the inflammatory process and are the key mediators of the innate immune response in humans. Similar to pro-apoptotic caspases, they are produced in an inactive form, and after an adequate stimulus is delivered via proper receptors, they are activated, and the activated inflammation complex (the so-called inflammasome) is formed [12].

## 2. Caspase-14

Caspase-14 is a unique member of the evolutionarily conserved family of cysteinyl aspartate-specific proteinases. The human caspase-14 gene is located on chromosome 19p13.1 and consists of seven exons. The native caspase-14 protein, with a molecular weight of 28 kDa, is not catalytically active [13]. Kallikrein-related peptidase-7 (KLK7) generates an activation intermediate comprising 20- and 8-kDa subunits by cleavage after Tyr178 of procaspase-14. This intermediate cleaves procaspase-14 at Asp146, generating mature and active caspase-14 [14].

It was suggested that caspase-14 was activated somewhere between the granular and cornified layers. Both the active and inactive forms were found in the total epidermal extract, whereas only the active form was present in the cornified layer [15]. Moreover, caspase-14 activation takes place simultaneously with the formation of the stratum corneum during embryonic development [16,17].

The identification of the substrates of caspase-14 has been a goal for researchers for many years. It is already known that in the case of in vitro conditions, proteolytically processed caspase-14 requires high concentrations of a kosmotropic salt (for example, sodium citrate) to be active [18].

Different substrate preferences are presented by murine and human caspase-14 under such conditions. The human one preferentially accommodates tryptophan or tyrosine in the S4 subsite, while the murine one—equally beta-branched and aromatic amino acids [18]. It is unknown whether those preferences demonstrated in vitro reflect the in vivo situation. Currently, profilaggrin is the only defined natural substrate for caspase-14.

Caspase-14 is an extraordinary member of the caspase family. Other representatives are present in numerous cells and tissues, while this one occurs only in several particular locations. It was observed only in mammals, mostly in the cornifying epithelia, Hassall’s bodies of the thymus gland, and in the forestomach of rodents [19,20]. As regards the epidermis, it is expressed in differentiating and cornifying layers and the hair follicles. It is absent in nail matrix keratinocytes, the noncornifying keratinocytes of sweat glands, and the epithelium of the oral cavity [19,21].

## 3. Caspase-14 Regulation

The transcriptional regulation of the caspase-14 gene has not been fully elucidated. Under laboratory conditions, caspase-14 is only expressed when keratinocytes are forced to differentiate by being grown post-confluently in high cell-density culture, a forced suspension culture, or by adding vitamin D3 [13,19,20,22]. Ca^2+^ added at high concentrations to the medium, a method often used to stimulate differentiation, did not stimulate caspase-14 expression [22]. Retinoids, at concentrations suppressing keratinocyte differentiation, downregulated caspase-14 expression [16,20]. Conversely, caspase-14 expression may be stimulated by polyphenols, e.g., epigallocatechin-3-gallate EGCG [23,24], especially if delivered via PEGylated liposomes [25]. Delphinidin is an anthocyanin and a potent antioxidant found in pigmented fruits and vegetables that may also stimulate epidermal cell differentiation with caspase-14 expression upregulation [26].

Sphingolipids are intracellular signal mediators in cell differentiation, growth inhibition, and apoptosis. High activity levels were particularly demonstrated in the case of the sphingoid base backbones of sphingolipids (sphingosine, sphinganine, and phytosphingosine) and their metabolites—N-acyl-sphingoid bases (ceramides). Firstly, ceramides were confirmed to be the stimulators of the upregulation of caspase-14 [27]. Subsequently, the mRNA of caspase-14 appeared to be stimulated by sphingoid bases and ceramides to increase intracellular caspase-14 protein level involving the specific inhibitors of mitogen-activated protein kinase, p38, and c-jun N-terminal protein kinase (JNK) [28].

The immune system interacts with numerous processes concerning cell biology. The balance between Th1- and Th2-type reactions provides stability, and fluctuations may lead to changes in the function of cells. It was shown that that the synthesis of caspase-14 was decreased in cultured human keratinocytes in response to Th2-associated cytokines. The addition of IL-4, IL-13, or both, resulted in a significant downregulation of caspase-14 protein production [29].

## 4. Caspase-14 and the Skin

Most caspases are expressed ubiquitously in all cells. Caspase-14 is an exception, as it is expressed only in particular cells. It was observed only in mammals, mostly in the cornifying epithelia [20]. In the epidermis, it is expressed in the differentiating and cornifying layers and hair follicles. In regards to ultrastructural analysis, the distribution of caspase-14 in the epidermis and hair follicles is conserved among several mammalian species [30,31]. In the granular layer, caspase-14 is associated with the nucleus, the keratohyalin granules, and the desmosomes, whereas in corneocytes, caspase-14 is present in the cytoplasm and connected to corneodesmosomes and nuclear remnants. Such results could suggest the role s caspase-14 in nuclear degradation during cornification, but nuclear degradation appeared unchanged in caspase-14-deficient mice [32].

Caspase-14 participates in skin barrier formation from the early embryonic stages. Its expression was noted from the embryonic day 14.5 [15] or 15.5 [33] and its processing from embryonic day 17.5 [33]. The cornified layer of the epidermis is also formed at those stages.

The main location in the epidermis suggests that caspase-14 could have a particular role there.

Skin is the largest organ of the human body. It plays an enormous role in protecting the body from dangerous external factors, e.g., chemical, physical injuries, UVB radiation, microorganisms, and, importantly in terrestrial species, water loss. The upper layer of the skin (epidermis) is the most important for this function, as it is the outer layer of the skin that is continuously renewed.

**Figure 1:**
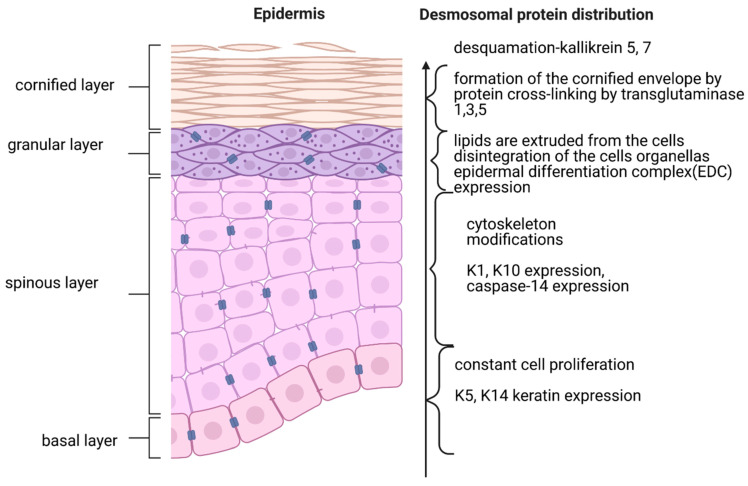
Epidermal desquamation process.

The lowest layer of the epidermis is called the basal layer, and it consists of proliferating keratinocytes. It is the layer where stem cells are located, enabling the cycle of continuous production of new keratinocytes and the desquamation of old ones. (Figure 1) These cells detach from the basement membrane and undergo multiple modifications leading to the final differentiation to transform into corneocytes. The transformation from the proliferating keratinocytes into corneocytes is called cornification. The process is well-organized and driven by multiple factors, especially transcriptional modifications in gene expression. Conversely, only a perfectly coordinated process leads to the final result, which is the development of an effective barrier. The premature death of the epithelial cells could lead to dysfunctional modifications in cellular structure or inflammation, thereby losing the primary role. Therefore, keratinocytes seem to have developed efficient anti-apoptotic and anti-necrotic mechanisms. NF-kB, the transcriptional factor conferring resistance to apoptosis, seems to play a crucial role in this process [34,35].

The spinous layer, granular layer, translucent layer, and cornified layer are located above the basal layer. When the epidermal cells move to the upper layers, they change their gene expression profile. They express keratins (K1 and K10) that are characteristic of differentiating keratinocytes. Previously, in the basal layer, they express K5 and K14 keratins, typical of proliferating keratinocytes. The process is performed due to transcriptional factors such as p63 [36,37]. A gene cluster called the epidermal differentiation complex (EDC) is another gene that starts to be expressed in the upper layers. It results in the production of such proteins as involucrin and loricrin in the upper layers.

The spinous layer is where cells improve their cytoskeletal keratin filament network. The interaction between adjacent cells occurs via desmosomes.

In the granular layer, the cells become more flattened and start to express particular proteins such as loricrin and profilaggrin accumulated in the keratohyalin granules. These granules are the reason for the name of the layer. Lipid production also takes place here.

A mechanically strong protective barrier is built due to several processes occurring between the granular and cornified layers.

The first step is the reorganization of cytoskeletal keratin filament into a strong network. The process of filament aggregation is dependent on filaggrin function. Its precursors are produced in the keratohyalin granules. Subsequently, they are dephosphorylated and proteolyzed to gain the final function [38]. Caspase-14 seems to be the effective enzyme for this proteolysis. After filaggrin completes its task, it undergoes further degradation into free amino acids, which is also driven by caspase-14. It was suggested that caspase-14 might either cleave filaggrin fragments and expose cleavage sites that may be recognized by other endo- and/or exopeptidases for further degradation, or it may be the activator of other cleaving endo- and/or exopeptidases [39,40]. The correct degradation of filaggrin into free amino acids was affected in caspase-14-deficient skin [30]. Loss-of-function mutations in the filaggrin gene (FLG) led to the development of dry skin disease, ichthyosis vulgaris in humans, and a similar neonatal skin disease in mice [41].

The second step involves the production of a cornified envelope on the peripheries of the cells. It is due to the proteins that are cross-linked by transglutaminases at the inner surface of the cytoplasmatic membrane (mostly small proline-rich proteins—SPRRs, and larger proteins like involucrin and loricrin). The transglutamination is performed by transglutaminases specific for the cornifying structure: transglutaminases 1, 3, and 5 [42].

The two elements are surrounded by a water-repelling coat that consists of lipids and natural moisturizing factors (NMFs). Caspase-14 seems to participate in the process of the construction of the modified cytoskeleton covered with a cornified envelope and in the formation of a lipid water-repelling coat. It cooperates in prosaposin processing together with mesotrypsin. Prosaposin is a precursor of four sphingolipid activator proteins (a-D) that are important in the lysosomal hydrolysis of sphingolipids. This process is essential for forming the lipid cover around the cornified envelope, which translates into the provision of the epidermal barrier [43].

The cells lose their organelles, such as the nucleus, and become flat and dead in the process of the final formation of the cornified layer.

All the above-mentioned factors contribute to the strong mechanics of the skin barrier. Corneocytes are eventually desquamated from the skin and replaced by newly formed ones [44]. The enzymatic process of shedding is dependent on kallikreins—serine proteases with tryptic or chymotryptic-like activity. Kallikreins 5 and 7 are involved and cleave corneodesmosome structural proteins, such as corneodesmosin, desmocollin, and desmoglein [45].

As described, the cornification process is quite a unique form of cell death. Seemingly, caspase-14 plays an important role in this process. However, no differences in the development of the skin barrier were seen in caspase-14-deficient mice. The skin of caspase-14-deficient mice was shinier and had larger scales and deeper skin lines [46]. However, the cornified envelope remained unchanged, which suggests that other factors might participate in the process.

## 5. Roles of Caspase-14

Processing profilaggrin, which is the precursor of filaggrin, is one of the well-defined roles of caspase-14. After profilaggrin is properly processed into filaggrin and then into hygroscopic amino acids, it acts as one of the elements of natural moisturizing factors (NMF), protecting the skin from water loss. It was shown that caspase-14 participated in both processes: profilaggrin into filaggrin and filaggrin into free amino acid degradation [47]. Therefore, caspase-14 plays a role in maintaining the skin barrier and protecting it from water loss. Filaggrin was detected in caspase-14-deficient mice, but it had a lower molecular weight, and its fragments were improperly accsumulated in the cornified layer [32,39,46]. This function of caspase-14 may be blocked by the infection of the epidermal cells with human papillomavirus 8 (HPV8) [48].

Water loss, UVB radiation, and other environmental stresses are dangerous for terrestrial species. Caspases play a role in the protection against all of them. The development of caspase-14-deficient mice revealed that the absence of caspase-14 caused sensitivity to UVB-induced photodamage and the apoptosis of the skin. It could be due to the UVB-filtering capacity of the stratum corneum that is reduced in caspase-14-deficient skin. It was demonstrated with higher levels of cyclobutane pyrimidine dimers that were detected presenting the molecular sign of UVB DNA damage [28].

The structure, role, and regulating factors of caspase-14 were described not only following in vitro research but also in vivo on human tissues. A caspase was suggested to have a large role in protecting the body from outer stress factors and providing skin cell production and destruction balance. Therefore, laboratory investigations were followed by clinical research for its role in the pathophysiology of proliferative and inflammatory conditions. Apparently, this protein plays an important role in the development of numerous neoplasms, skin diseases, and other health conditions. It was also considered as a target molecule for potential innovative methods of treatment.

## 6. Caspase-14 and Cancer

The dysregulation of the expression of caspase-14 was described in neoplastic human cells and in several cancer cell lines. (Table 1).

An interesting introduction to the analysis of caspase-14 in neoplastic cells was presented in 2005 in research, including few neoplasm types and caspase-14 testing via the immunohistochemical examination of tissues. The expression of caspase-14 was significantly reduced in the tested material from the cervical, ovarian, and colon cancer specimens. The decrease in caspase-14 immunopositivity correlated with the histologic progression of cervical cancer. Lower caspase-14 expression was associated with the advanced clinical stage in ovarian cancer and with shorter overall survival among ovarian cancer patients with serous tumors. Contrary to cervical, ovarian, and colon cancers, caspase-14 expression was increased in ductal breast carcinoma in situ and invasive cancers compared to the normal mammary epithelium.

As regards localized gastric cancers, caspase-14 immunostaining was significantly lower in poorly differentiated tumors compared to well-differentiated ones. Lower caspase-14 expression correlated with shorter overall survival in patients with T3N0M0 stage gastric cancer [51]. Pilot research performed in 2005 was an introduction to further extensive clinical observations performed by other researchers on numerous cancer samples derived from a variety of tissues and organs.

Caspase-14 is not expressed in normal salivary glands. However, an aberrant expression was found in a subfraction of carcinomas (32%). Caspase-14 staining was not associated with tumor dedifferentiation, GATA3 expression, or the amplification of gene locus 19p13 [52]. Another research on salivary gland malignancies showed that the human salivary gland cancer cell line (HSG) was transiently transfected with full-length human caspase-14 cDNA. It revealed the inhibition of growth and viability. It also demonstrated the reduced tumorigenicity, probably due to the reduced vascularization of the tumor. The research was designed to estimate the possibilities of gene therapy with the caspase-14 gene [53].

Research on vulvar neoplasia revealed that the expression of caspase-14 was significantly reduced in the precancerous status and in invasive squamous cell carcinoma of the vulva. It was demonstrated that its expression was upregulated after exposure to black raspberry extract [49].

Lung adenocarcinoma was the target cancer that was also analyzed in correlation with caspase-14. Caspase-14 was identified as a protein interacting with apoptosis inducing factor (AIF). AIF is not only a protein inducing apoptosis but also an essential mitochondrial flavoprotein. After exposure to genotoxic factors such as cisplatin, adriamycin, staurosporine, capecitabine, or etoposide, it is directly transported from the mitochondrion to the cell nucleus, where it starts the process of polymerase degradation, DNA fragmentation, chromatin condensation, and the formation of the micronucleus, i.e., it triggers apoptosis.

It was shown that lung adenocarcinoma overexpressed caspase-14. Moreover, the elevated levels correlated with poor prognosis for patients. Under in vitro conditions, the overexpression of caspase-14 led to resistance to cisplatin treatment. It was suggested that the inhibition of caspase-14 could reduce the resistance to chemotherapy via the AIF pathway [50,61,62].

Oral cavity squamous carcinoma presented the reduced expression of caspase-14 in 47% of tested tumors and was not related to keratinization, tumor differentiation, or HPV infection [63].

Skin cancer is a neoplasm that is highly linked to chronic UV exposure. In the murine model, the expression of caspase-14 mRNA was reduced in squamous cell carcinoma induced by UVB. The global gene expression profiles of normal murine epidermis and UV-induced squamous cell carcinomas (SCCs) in mice showed an 8-fold lower expression of caspase-14 in squamous cell carcinoma [17]. In the case of skin cancer, the transfection of human epidermoid cancer cell lines (A431) with the caspase-14-expressing pCMV vector led to the reduction of the growth and tumorigenicity of cells [64]. The overexpression of caspase-14 significantly decreased cell proliferation, delayed tumor growth, and induced the expression of three squamous differentiation markers in A431 cells, epidermal cancer cell line model cells [65].

The above research was performed in epidermoid cell lines or murine models, but not in skin cancer or human cells in vivo.

Melanoma is a malignancy arising from melanocytes. Therefore, it cannot be analyzed as a planoepithelial malignancy. The expression of caspase-14 mRNA was confirmed in 97% of melanoma cells. Moreover, its reduced level was related to sensitivity to treatment with capecitabine, cisplatin, and radiotherapy [54].

## 7. Caspase-14 and Other Diseases

Research concerning the barrier functions was conducted, as caspase does not only participate in coordinating balance between differentiation, cell growth, and death in the skin, but it also seems to be a potent player in the protection from environmental factors.

It was demonstrated that retinal cells—pericytes (PCs), endothelial cells (ECs), astrocytes (ACs), choroidal endothelial cells, and retinal pigment epithelium (RPE) cells expressed caspase-14. Diabetic retinopathy was associated with the upregulation and/or activation of caspase-14, particularly in the retinal vasculature. High glucose-induced the elevation of caspase-14 level in retinal vascular cells [55]. Other authors suggested that caspase-14 contributed to RPE cell barrier disruption under hyperglycemic conditions and might be important in the development of diabetic macular edema [56].

As shown with real-time reverse-transcription PCR, the expression level of caspase-14 mRNA in the cholesteatoma epithelium was significantly higher than in the normal external auditory canal epithelium. Caspase-14 protein was detected both in the normal external auditory canal and cholesteatoma, but its expression was shown to be greater in cholesteatoma on Western blot analysis [66].

## 8. Caspase-14 and Skin Diseases

Psoriasis is an inflammatory disease of a complex etiology. Histopathologically, it is characterized by the uncontrolled proliferation of keratinocytes and impaired cornification. It leads to the aberrant presence of cell organelles, especially the nucleus in the cornified layer. It is called parakeratosis. It was demonstrated that caspase-14 was downregulated in psoriatic lesions but not in the healthy skin around [18,57,65].

Although caspase-14 is absent in parakeratotic regions, it is probably not the cause of the development of parakeratotic plaques, as caspase-14-deficient mice did not show spontaneous parakeratosis [39]. Impaired cornification was suggested in caspase-14-deficient mice, as incompletely cornified cells were found in the transitional zone between the granular and the cornified layers (parakeratosis-like sign). Moreover, the exposure of the skin of those mice to acetone or imiquimod (inducing psoriasis-like lesions) led to parakeratosis more commonly than in the control group of mice [58].

Conversely, the topical treatment of psoriatic lesions with a vitamin D3 analog resulted in the reduction of the psoriatic phenotype and a higher caspase-14 expression in the parakeratotic lesions [20].

As caspase-14 plays an essential role in the epidermal skin barrier, it has to be involved in the development of skin disease, with the most important symptom being epidermal skin barrier dysfunction, i.e., atopic dermatitis. The expression level of caspase-14, similarly to pyrrolidone carboxylic acids (PCA), which are natural moisturizing factors, is significantly decreased in inflammatory lesions compared to non-lesional areas of the skin affected by atopic dermatitis. Moreover, its level correlated with disease severity assessed with the index of the area affected and severity [59]. However, no effect of genetic variants of the CASP14 was determined as regards the risk of xerosis or atopic dermatitis [67].

Eczema is another skin disease that affects the skin barrier. The expression of caspase-14 is decreased in the lesional and non-lesional skin of patients with chronic hand eczema. It was found to be correlated with increased pH value, decreased water content, increased TEWL (transepidermal water loss), and the impaired integrity of the cornified layer of the epidermis [60].

The hyperkeratosis of the skin is the main feature of callosity. Research conducted on human epidermis samples revealed that the overexpression of caspase-14 along with involucrin and filaggrin might play a role in the development of this dermatological condition. Obviously, they are the markers of the cornification of the epidermis [68].

The placenta is an extraordinary organ that constitutes a tight connection between the mother and the baby, at the same time providing a barrier between those two. It allows the influx of beneficial factors, such as energy, immunoglobulins, hormones, and prevents the harmful ones from entering, e.g., toxins or microorganisms.

The human placenta consists of the cytotrophoblast and syncytiotrophoblast. The latter provides a barrier between the maternal and fetal tissues. The disruption in trophoblast development and differentiation may lead to impaired fetal growth and eclampsia.

It was demonstrated in 2005 that caspase-14 was expressed in the human placenta. The expression was higher in first-trimester placentas than in placentas at term [69].

Research on BeWo cell lines (the choriocarcinoma-derived cell used as a model of trophoblast) showed that caspase-14 seemed to have an opposite function in that location than in the human epidermis. The suppression of caspase-14 in those cells led to the increased transcription of KLF4, hCG, and cytokeratin-18, which are increased during normal trophoblast differentiation. It was suggested that caspase-14 had an effect on that pathway of differentiation [70,71].

## 9. Summary

Control over death and life is the goal of medical science. On the one hand, we try to discover the secret of long and healthy life and achieve it. On the other hand, we want to gain control over neoplastic diseases with cells that have escaped processes leading to cell death. Research concerning the molecular pathways of cell death is crucial in both cases. Numerous cells and tissues may be used as life-derived models of different processes in the human body. Healthy liver cells are the model of tissue that may be regenerated after injury in a well-controlled process. Neoplastic cells are the model of uncontrolled proliferation, omitting the control points of the cell cycle and unlimited growth. The epidermal cells are an extraordinary example of cells that have the ability to regenerate to a certain extent, have a unique planned death program, i.e., cornification, and have effectively omitted apoptosis so as not to lose the primary function.

Understanding each of the models brings researchers closer to understanding the life and death cycle of cells and offers a potential chance for clinical applications.

Caspase-14 is an example of a protein that may play a crucial role in this process. It belongs to the apoptotic family, being a non-apoptotic member. It plays an enormous role in providing the environmental barrier for the body. Moreover, its expression was noted in many diseases, and some attempts of using this knowledge have already been made. Neoplasms seem to present different patterns of expression of caspase-14. In some neoplasms, its level is increased, and in others, it is decreased. Therefore, we cannot conclude that any of these tendencies might be generalized to all malignancies. However, knowledge about the trends in particular cancers could be useful in estimating the prognosis and, potentially, drug susceptibility as presented in the case of adenocarcinoma [50]. Conversely, some malignancies, like salivary gland carcinoma, may be cases in which caspase-14 could be the target molecule for treatment. It was presented that the exogenous expression of caspase-14 reduced the tumorigenicity of the model cell lines of salivary gland carcinoma. In such a case, more extensive in vivo research seems to be promising and of great interest.

As regards skin conditions, in which caspase-14 expression levels are usually decreased, performing more research appeared interesting for the authors, especially concerning the potential possibilities of the stimulation of molecule upregulation as a potential treatment option.

Even if caspase does not prove to be the ultimate anchor point for therapy—following it may give us a better perspective of the homeostasis and balance in various tissues.

The authors consider this molecule as a protein that needs to be analyzed in many more clinical, pathophysiological entities, not only in dermatology but in oncology as well.

## Figures and Tables

**Table 1:** Caspase-14 in diseases.

Disease	Result	Author
Cancer of the vulva	The expression was decreased in the precancerous status and squamous cell cancer; The exposure to black raspberry extract induced caspase-14 expression.	[49]
Lung adenocarcinoma	The expression was increased; The increased expression correlated with a poorer prognosis; In vitro increased expression correlated with the resistance to treatment with cisplatin.	[50]
Cervical cancer	Expression decreased; The decreased expression correlated with more advanced staging.	[51]
Ovarian cancer	Expression decreased; The decreased expression correlated with advanced clinical stages and shorter overall survival rates in patients with serous cancers.	[51]
Colon cancer	Expression decreased.	[51]
Gastric cancer	In localized gastric cancer, the expression was decreased in poorly differentiated cancer; Patients with stage T3N0M0 tumors that contained higher levels of caspase-14 had significantly longer overall survival.	[51]
Breast cancer	Increased expression in ductal cancer—both in situ and infiltrating.	[51]
Salivary gland cancer	Physiologically, no expression in the salivary gland; Expression in 32% of tested salivary gland adenocarcinomas.	[52]
	In human salivary gland cancer cell line HSG was transfected with caspase-14: cell growth and viability were inhibited, a significant reduction of tumorigenicity was observed together with a decrease in blood vessel formation.	[53]
Malignant melanoma	Caspase-14 was expressed in 97% of malignant melanoma cells, but only in 70% of dermal nevi; Lower expression of caspase-14 correlated with better response to treatment with chemotherapy and radiotherapy.	[54]
Diabetic retinopathy	Retinal cells—pericytes (PCs), endothelial cells (ECs), astrocytes (ACs), choroidal endothelial cells, and retinal pigment epithelium (RPE) cells expressed caspase-14; Diabetic retinopathy was associated with the upregulation and/or activation of caspase-14, particularly in the retinal vasculature.	[55,56]
Psoriasis	Decreased only in lesions, but not in the healthy skin around.	[20,57,58]
Atopic dermatitis	Significantly decreased in the inflammatory lesions compared to non-lesional skin; Its level correlated with disease severity.	[59]
Chronic hand eczema	Decreased in lesional and non-lesional skin.	[60]

## Data Availability

Not applicable.
